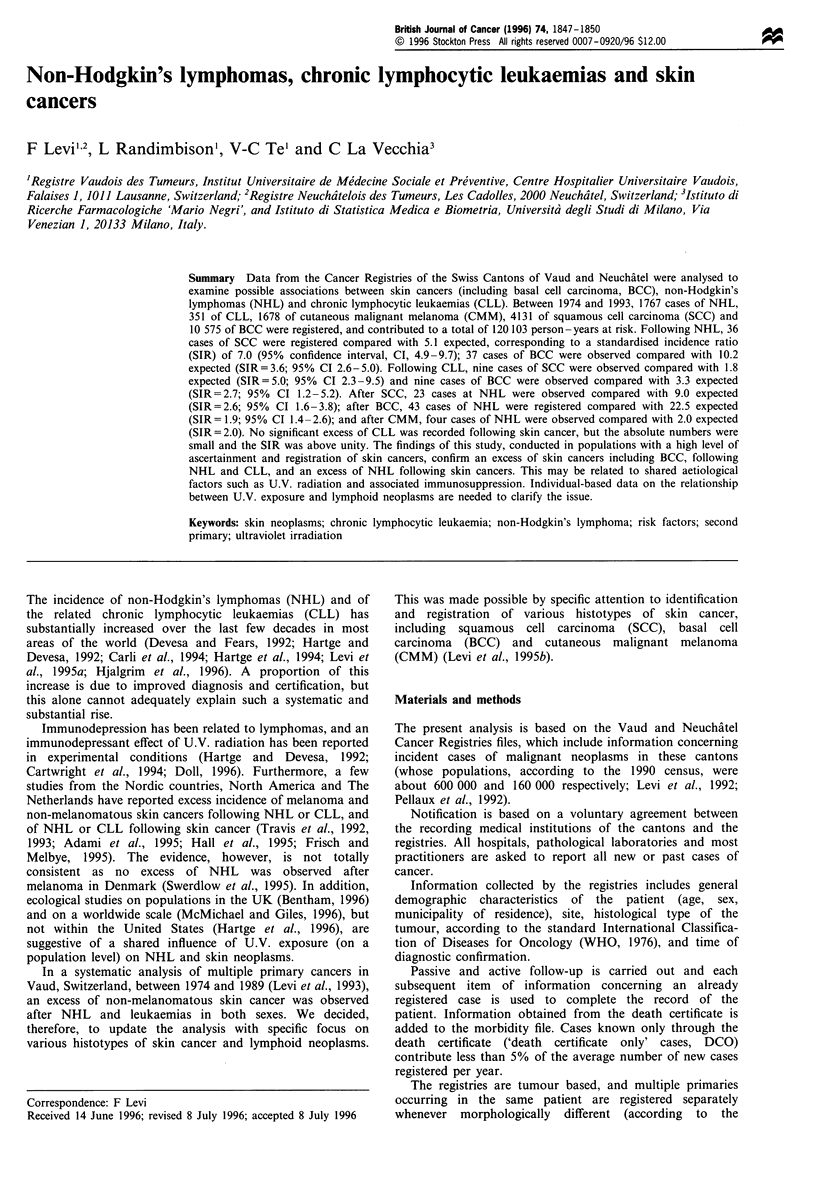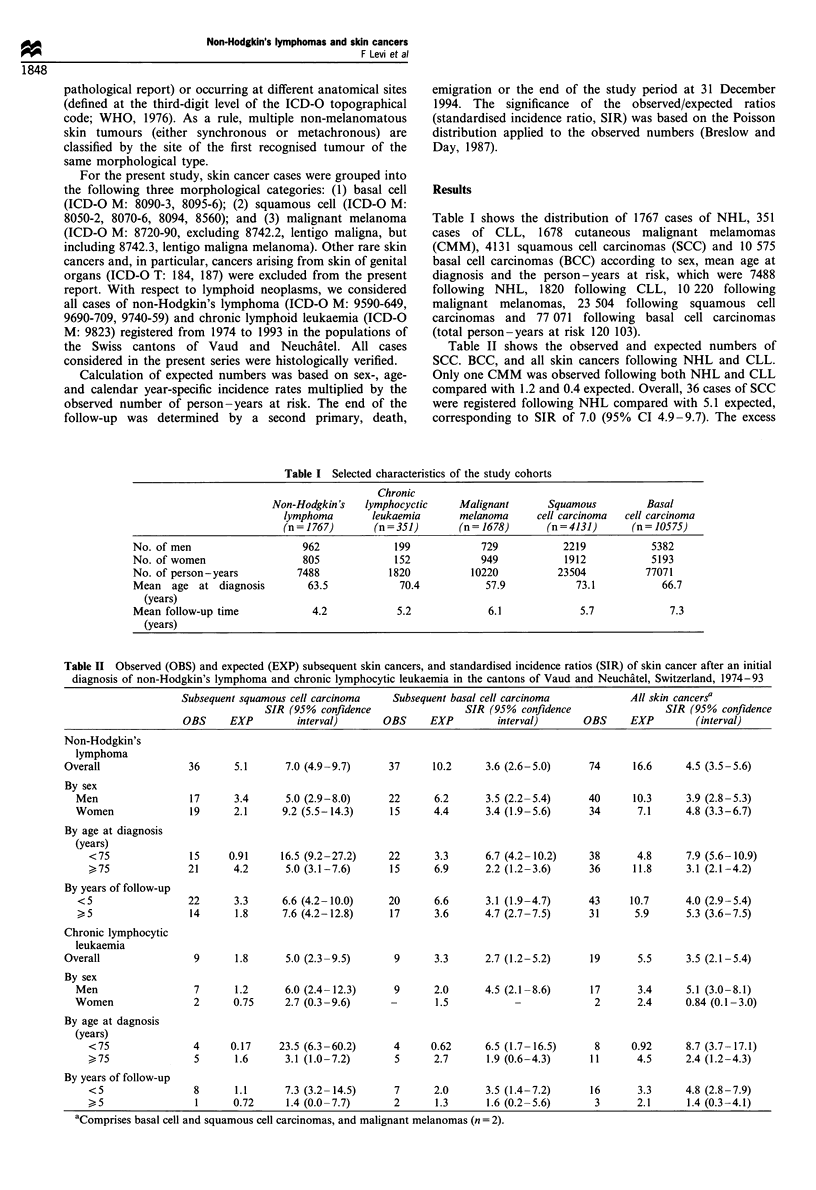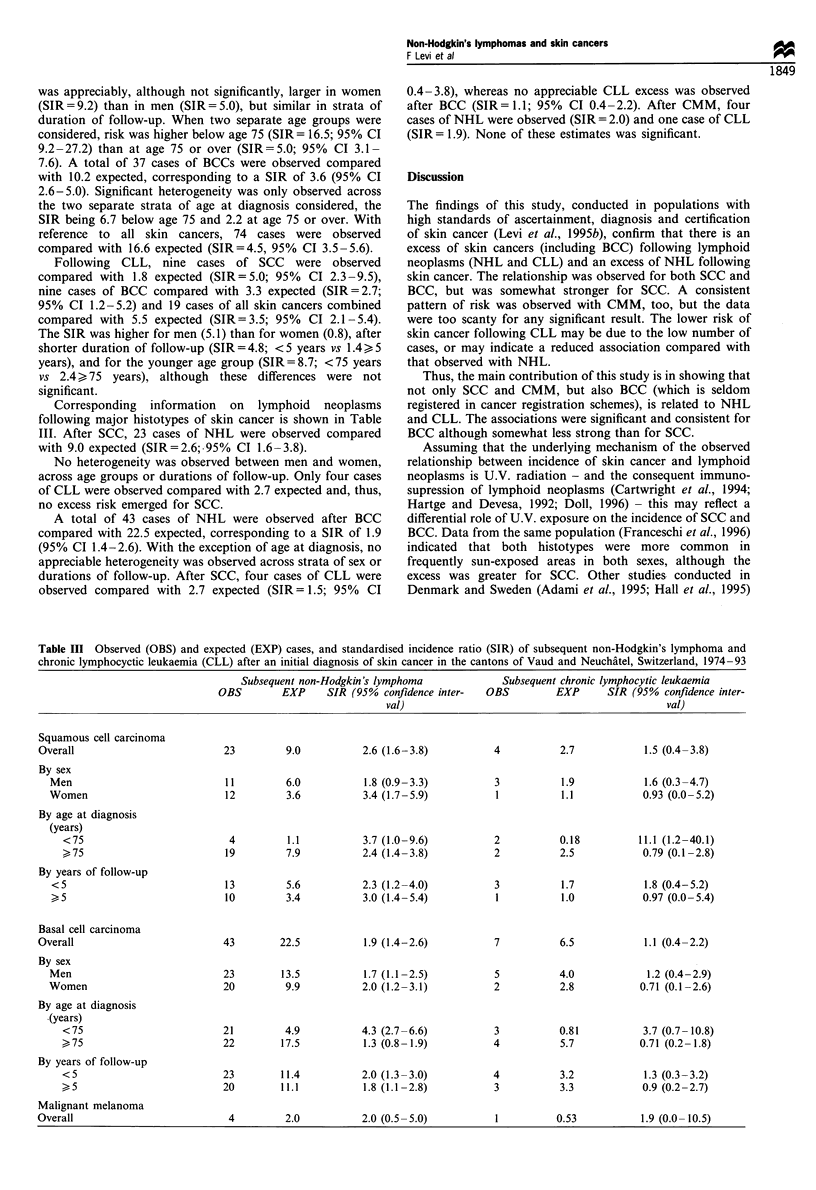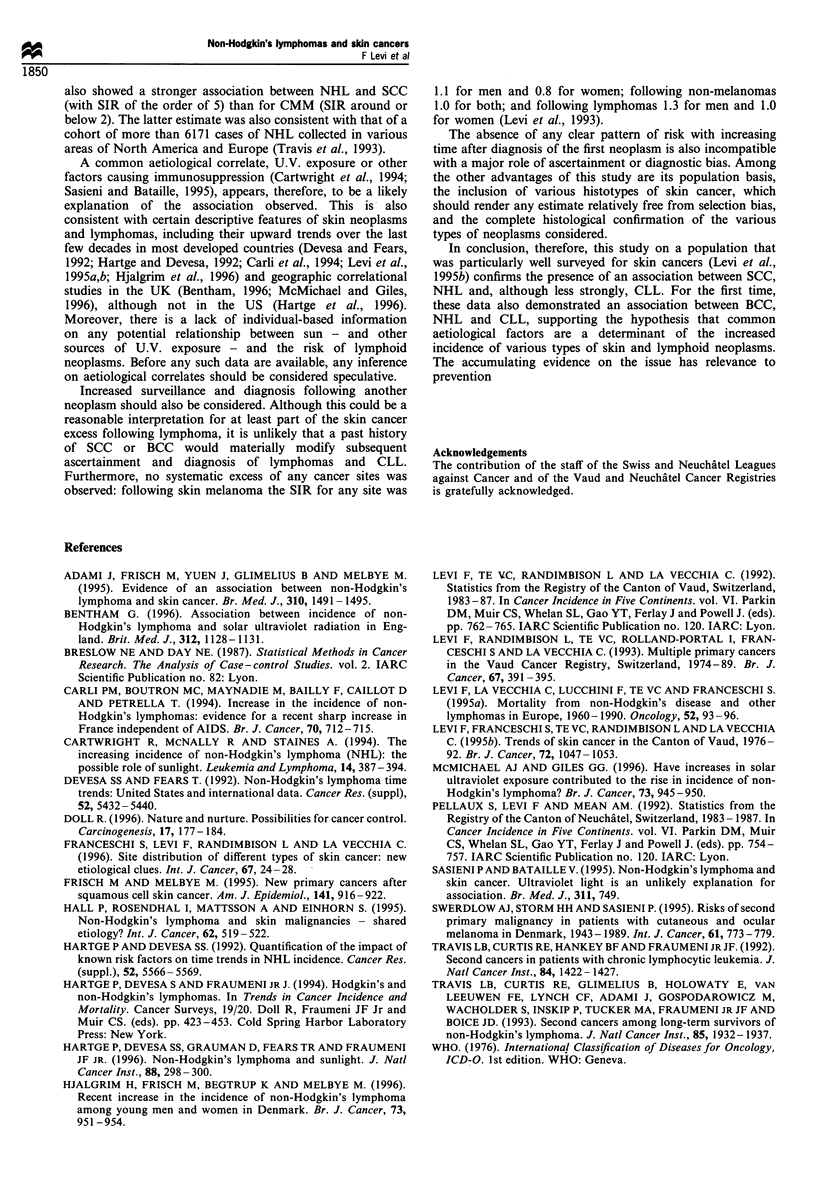# Non-Hodgkin's lymphomas, chronic lymphocytic leukaemias and skin cancers.

**DOI:** 10.1038/bjc.1996.642

**Published:** 1996-12

**Authors:** F. Levi, L. Randimbison, V. C. Te, C. La Vecchia

**Affiliations:** Registre Vaudois des Tumeurs, Institut Universitaire de Médecine Sociale et Préventive, Centre Hospitalier Universitaire Vaudois, Lausanne, Switzerland.

## Abstract

Data from the Cancer Registries of the Swiss Cantons of Vaud and Neuchâtel were analysed to examine possible associations between skin cancers (including basal cell carcinoma, BCC), non-Hodgkin's lymphomas (NHL) and chronic lymphocytic leukaemias (CLL). Between 1974 and 1993, 1767 cases of NHL, 351 of CLL, 1678 of cutaneous malignant melanoma (CMM), 4131 of squamous cell carcinoma (SCC) and 10575 of BCC were registered, and contributed to a total of 120103 person-years at risk. Following NHL, 36 cases of SCC were registered compared with 5.1 expected, corresponding to a standardised incidence ratio (SIR) of 7.0 (95% confidence interval, CI, 4.9-9.7); 37 cases of BCC were observed compared with 10.2 expected (SIR = 3.6; 95% CI 2.6-5.0). Following CLL, nine cases of SCC were observed compared with 1.8 expected (SIR = 5.0; 95% CI 2.3-9.5) and nine cases of BCC were observed compared with 3.3 expected (SIR = 2.7; 95% CI 1.2-5.2). After SCC, 23 cases at NHL were observed compared with 9.0 expected (SIR = 2.6; 95% CI 1.6-3.8); after BCC, 43 cases of NHL were registered compared with 22.5 expected (SIR = 1.9; 95% CI 1.4-2.6); and after CMM, four cases of NHL were observed compared with 2.0 expected (SIR = 2.0). No significant excess of CLL was recorded following skin cancer, but the absolute numbers were small and the SIR was above unity. The findings of this study, conducted in populations with a high level of ascertainment and registration of skin cancers, confirm an excess of skin cancers including BCC, following NHL and CLL, and an excess of NHL following skin cancers. This may be related to shared aetiological factors such as U.V. radiation and associated immunosuppression. Individual-based data on the relationship between U.V. exposure and lymphoid neoplasms are needed to clarify the issue.


British Journal of Cancer (1996) 74, 1847–1850
© 1996 Stockton Press All rights reserved 0007–0920/96 $12.00

### Non-Hodgkin's lymphomas, chronic lymphocytic leukaemias and skin cancers

F Levi[1,2], L Randimbison[1], V-C Te[1] and C La Vecchia[3]

*[1]Registre Vaudois des Tumeurs, Institut Universitaire de Médecine Sociale et Préventive, Centre Hospitalier Universitaire Vaudois, Falaises 1, 1011 Lausanne, Switzerland; [2]Registre Neuchâtelois des Tumeurs, Les Cadolles, 2000 Neuchâtel, Switzerland; [3]Istituto di Ricerche Farmacologiche 'Mario Negri', and Istituto di Statistica Medica e Biometria, Università degli Studi di Milano, Via Venezian 1, 20133 Milano, Italy.*

**Summary** Data from the Cancer Registries of the Swiss Cantons of Vaud and Neuchâtel were analysed to examine possible associations between skin cancers (including basal cell carcinoma, BCC), non-Hodgkin's lymphomas (NHL) and chronic lymphocytic leukaemias (CLL). Between 1974 and 1993, 1767 cases of NHL, 351 of CLL, 1678 of cutaneous malignant melanoma (CMM), 4131 of squamous cell carcinoma (SCC) and 10 575 of BCC were registered, and contributed to a total of 120 103 person–years at risk. Following NHL, 36 cases of SCC were registered compared with 5.1 expected, corresponding to a standardised incidence ratio (SIR) of 7.0 (95% confidence interval, CI, 4.9–9.7); 37 cases of BCC were observed compared with 10.2 expected (SIR = 3.6; 95% CI 2.6–5.0). Following CLL, nine cases of SCC were observed compared with 1.8 expected (SIR = 5.0; 95% CI 2.3–9.5) and nine cases of BCC were observed compared with 3.3 expected (SIR = 2.7; 95% CI 1.2–5.2). After SCC, 23 cases at NHL were observed compared with 9.0 expected (SIR = 2.6; 95% CI 1.6–3.8); after BCC, 43 cases of NHL were registered compared with 22.5 expected (SIR = 1.9; 95% CI 1.4–2.6); and after CMM, four cases of NHL were observed compared with 2.0 expected (SIR = 2.0). No significant excess of CLL was recorded following skin cancer, but the absolute numbers were small and the SIR was above unity. The findings of this study, conducted in populations with a high level of ascertainment and registration of skin cancers, confirm an excess of skin cancers including BCC, following NHL and CLL, and an excess of NHL following skin cancers. This may be related to shared aetiological factors such as U.V. radiation and associated immunosuppression. Individual-based data on the relationship between U.V. exposure and lymphoid neoplasms are needed to clarify the issue.

**Keywords:** skin neoplasms; chronic lymphocytic leukaemia; non-Hodgkin's lymphoma; risk factors; second primary; ultraviolet irradiation

The incidence of non-Hodgkin's lymphomas (NHL) and of the related chronic lymphocytic leukaemias (CLL) has substantially increased over the last few decades in most areas of the world (Devesa and Fears, 1992; Hartge and Devesa, 1992; Carli *et al.*, 1994; Hartge *et al.*, 1994; Levi *et al.*, 1995*a*; Hjalgrim *et al.*, 1996). A proportion of this increase is due to improved diagnosis and certification, but this alone cannot adequately explain such a systematic and substantial rise.

Immunodepression has been related to lymphomas, and an immunodepressant effect of U.V. radiation has been reported in experimental conditions (Hartge and Devesa, 1992; Cartwright *et al.*, 1994; Doll, 1996). Furthermore, a few studies from the Nordic countries, North America and The Netherlands have reported excess incidence of melanoma and non-melanomatous skin cancers following NHL or CLL, and of NHL or CLL following skin cancer (Travis *et al.*, 1992, 1993; Adami *et al.*, 1995; Hall *et al.*, 1995; Frisch and Melbye, 1995). The evidence, however, is not totally consistent as no excess of NHL was observed after melanoma in Denmark (Swerdlow *et al.*, 1995). In addition, ecological studies on populations in the UK (Bentham, 1996) and on a worldwide scale (McMichael and Giles, 1996), but not within the United States (Hartge *et al.*, 1996), are suggestive of a shared influence of U.V. exposure (on a population level) on NHL and skin neoplasms.

In a systematic analysis of multiple primary cancers in Vaud, Switzerland, between 1974 and 1989 (Levi *et al.*, 1993), an excess of non-melanomatous skin cancer was observed after NHL and leukaemias in both sexes. We decided, therefore, to update the analysis with specific focus on various histotypes of skin cancer and lymphoid neoplasms. This was made possible by specific attention to identification and registration of various histotypes of skin cancer, including squamous cell carcinoma (SCC), basal cell carcinoma (BCC) and cutaneous malignant melanoma (CMM) (Levi *et al.*, 1995*b*).

Correspondence: F Levi
Received 14 June 1996; revised 8 July 1996; accepted 8 July 1996

### Materials and methods

The present analysis is based on the Vaud and Neuchâtel Cancer Registries files, which include information concerning incident cases of malignant neoplasms in these cantons (whose populations, according to the 1990 census, were about 600 000 and 160 000 respectively; Levi *et al.*, 1992; Pellaux *et al.*, 1992).

Notification is based on a voluntary agreement between the recording medical institutions of the cantons and the registries. All hospitals, pathological laboratories and most practitioners are asked to report all new or past cases of cancer.

Information collected by the registries includes general demographic characteristics of the patient (age, sex, municipality of residence), site, histological type of the tumour, according to the standard International Classification of Diseases for Oncology (WHO, 1976), and time of diagnostic confirmation.

Passive and active follow-up is carried out and each subsequent item of information concerning an already registered case is used to complete the record of the patient. Information obtained from the death certificate is added to the morbidity file. Cases known only through the death certificate ('death certificate only' cases, DCO) contribute less than 5% of the average number of new cases registered per year.

The registries are tumour based, and multiple primaries occurring in the same patient are registered separately whenever morphologically different (according to the pathological report) or occurring at different anatomical sites (defined at the third-digit level of the ICD-O topographical code; WHO, 1976). As a rule, multiple non-melanomatous skin tumours (either synchronous or metachronous) are classified by the site of the first recognised tumour of the same morphological type.

For the present study, skin cancer cases were grouped into the following three morphological categories: (1) basal cell (ICD-O M: 8090-3, 8095-6); (2) squamous cell (ICD-O M: 8050-2, 8070-6, 8094, 8560); and (3) malignant melanoma (ICD-O M: 8720-90, excluding 8742.2, lentigo maligna, but including 8742.3, lentigo maligna melanoma). Other rare skin cancers and, in particular, cancers arising from skin of genital organs (ICD-O T: 184, 187) were excluded from the present report. With respect to lymphoid neoplasms, we considered all cases of non-Hodgkin's lymphoma (ICD-O M: 9590-649, 9690-709, 9740-59) and chronic lymphoid leukaemia (ICD-O M: 9823) registered from 1974 to 1993 in the populations of the Swiss cantons of Vaud and Neuchâtel. All cases considered in the present series were histologically verified.

Calculation of expected numbers was based on sex-, age- and calendar year-specific incidence rates multiplied by the observed number of person–years at risk. The end of the follow-up was determined by a second primary, death, emigration or the end of the study period at 31 December 1994. The significance of the observed/expected ratios (standardised incidence ratio, SIR) was based on the Poisson distribution applied to the observed numbers (Breslow and Day, 1987).

### Results

Table I shows the distribution of 1767 cases of NHL, 351 cases of CLL, 1678 cutaneous malignant melamomas (CMM), 4131 squamous cell carcinomas (SCC) and 10 575 basal cell carcinomas (BCC) according to sex, mean age at diagnosis and the person–years at risk, which were 7488 following NHL, 1820 following CLL, 10 220 following malignant melanomas, 23 504 following squamous cell carcinomas and 77 071 following basal cell carcinomas (total person–years at risk 120 103).

Table II shows the observed and expected numbers of SCC. BCC, and all skin cancers following NHL and CLL. Only one CMM was observed following both NHL and CLL compared with 1.2 and 0.4 expected. Overall, 36 cases of SCC were registered following NHL compared with 5.1 expected, corresponding to SIR of 7.0 (95% CI 4.9–9.7). The excess

**Table I** Selected characteristics of the study cohorts

| | *Non-Hodgkin's lymphoma (n=1767)* | *Chronic lymphocyctic leukaemia (n=351)* | *Malignant melanoma (n=1678)* | *Squamous cell carcinoma (n=4131)* | *Basal cell carcinoma (n=10575)* |
|---|---|---|---|---|---|
| No. of men | 962 | 199 | 729 | 2219 | 5382 |
| No. of women | 805 | 152 | 949 | 1912 | 5193 |
| No. of person–years | 7488 | 1820 | 10220 | 23504 | 77071 |
| Mean age at diagnosis (years) | 63.5 | 70.4 | 57.9 | 73.1 | 66.7 |
| Mean follow-up time (years) | 4.2 | 5.2 | 6.1 | 5.7 | 7.3 |

**Table II** Observed (OBS) and expected (EXP) subsequent skin cancers, and standardised incidence ratios (SIR) of skin cancer after an initial diagnosis of non-Hodgkin's lymphoma and chronic lymphocytic leukaemia in the cantons of Vaud and Neuchâtel, Switzerland, 1974–93

| | *Subsequent squamous cell carcinoma* | | | *Subsequent basal cell carcinoma* | | | *All skin cancers[a]* | | |
|---|---|---|---|---|---|---|---|---|---|
| | *OBS* | *EXP* | *SIR (95% confidence interval)* | *OBS* | *EXP* | *SIR (95% confidence interval)* | *OBS* | *EXP* | *SIR (95% confidence (interval)* |
| Non-Hodgkin's lymphoma | | | | | | | | | |
| Overall | 36 | 5.1 | 7.0 (4.9–9.7) | 37 | 10.2 | 3.6 (2.6–5.0) | 74 | 16.6 | 4.5 (3.5–5.6) |
| By sex | | | | | | | | | |
| Men | 17 | 3.4 | 5.0 (2.9–8.0) | 22 | 6.2 | 3.5 (2.2–5.4) | 40 | 10.3 | 3.9 (2.8–5.3) |
| Women | 19 | 2.1 | 9.2 (5.5–14.3) | 15 | 4.4 | 3.4 (1.9–5.6) | 34 | 7.1 | 4.8 (3.3–6.7) |
| By age at diagnosis (years) | | | | | | | | | |
| <75 | 15 | 0.91 | 16.5 (9.2–27.2) | 22 | 3.3 | 6.7 (4.2–10.2) | 38 | 4.8 | 7.9 (5.6–10.9) |
| ⩾75 | 21 | 4.2 | 5.0 (3.1–7.6) | 15 | 6.9 | 2.2 (1.2–3.6) | 36 | 11.8 | 3.1 (2.1–4.2) |
| By years of follow-up | | | | | | | | | |
| <5 | 22 | 3.3 | 6.6 (4.2–10.0) | 20 | 6.6 | 3.1 (1.9–4.7) | 43 | 10.7 | 4.0 (2.9–5.4) |
| ⩾5 | 14 | 1.8 | 7.6 (4.2–12.8) | 17 | 3.6 | 4.7 (2.7–7.5) | 31 | 5.9 | 5.3 (3.6–7.5) |
| Chronic lymphocytic leukaemia | | | | | | | | | |
| Overall | 9 | 1.8 | 5.0 (2.3–9.5) | 9 | 3.3 | 2.7 (1.2–5.2) | 19 | 5.5 | 3.5 (2.1–5.4) |
| By sex | | | | | | | | | |
| Men | 7 | 1.2 | 6.0 (2.4–12.3) | 9 | 2.0 | 4.5 (2.1–8.6) | 17 | 3.4 | 5.1 (3.0–8.1) |
| Women | 2 | 0.75 | 2.7 (0.3–9.6) | – | 1.5 | – | 2 | 2.4 | 0.84 (0.1–3.0) |
| By age at dagnosis (years) | | | | | | | | | |
| <75 | 4 | 0.17 | 23.5 (6.3–60.2) | 4 | 0.62 | 6.5 (1.7–16.5) | 8 | 0.92 | 8.7 (3.7–17.1) |
| ⩾75 | 5 | 1.6 | 3.1 (1.0–7.2) | 5 | 2.7 | 1.9 (0.6–4.3) | 11 | 4.5 | 2.4 (1.2–4.3) |
| By years of follow-up | | | | | | | | | |
| <5 | 8 | 1.1 | 7.3 (3.2–14.5) | 7 | 2.0 | 3.5 (1.4–7.2) | 16 | 3.3 | 4.8 (2.8–7.9) |
| ⩾5 | 1 | 0.72 | 1.4 (0.0–7.7) | 2 | 1.3 | 1.6 (0.2–5.6) | 3 | 2.1 | 1.4 (0.3–4.1) |

[a]Comprises basal cell and squamous cell carcinomas, and malignant melanomas ($n=2$).

was appreciably, although not significantly, larger in women (SIR = 9.2) than in men (SIR = 5.0), but similar in strata of duration of follow-up. When two separate age groups were considered, risk was higher below age 75 (SIR = 16.5; 95% CI 9.2–27.2) than at age 75 or over (SIR = 5.0; 95% CI 3.1–7.6). A total of 37 cases of BCCs were observed compared with 10.2 expected, corresponding to a SIR of 3.6 (95% CI 2.6–5.0). Significant heterogeneity was only observed across the two separate strata of age at diagnosis considered, the SIR being 6.7 below age 75 and 2.2 at age 75 or over. With reference to all skin cancers, 74 cases were observed compared with 16.6 expected (SIR = 4.5, 95% CI 3.5–5.6).

Following CLL, nine cases of SCC were observed compared with 1.8 expected (SIR = 5.0; 95% CI 2.3–9.5), nine cases of BCC compared with 3.3 expected (SIR = 2.7; 95% CI 1.2–5.2) and 19 cases of all skin cancers combined compared with 5.5 expected (SIR = 3.5; 95% CI 2.1–5.4). The SIR was higher for men (5.1) than for women (0.8), after shorter duration of follow-up (SIR = 4.8; <5 years *vs* 1.4⩾5 years), and for the younger age group (SIR = 8.7; <75 years *vs* 2.4⩾75 years), although these differences were not significant.

Corresponding information on lymphoid neoplasms following major histotypes of skin cancer is shown in Table III. After SCC, 23 cases of NHL were observed compared with 9.0 expected (SIR = 2.6; 95% CI 1.6–3.8).

No heterogeneity was observed between men and women, across age groups or durations of follow-up. Only four cases of CLL were observed compared with 2.7 expected and, thus, no excess risk emerged for SCC.

A total of 43 cases of NHL were observed after BCC compared with 22.5 expected, corresponding to a SIR of 1.9 (95% CI 1.4–2.6). With the exception of age at diagnosis, no appreciable heterogeneity was observed across strata of sex or durations of follow-up. After SCC, four cases of CLL were observed compared with 2.7 expected (SIR = 1.5; 95% CI 0.4–3.8), whereas no appreciable CLL excess was observed after BCC (SIR = 1.1; 95% CI 0.4–2.2). After CMM, four cases of NHL were observed (SIR = 2.0) and one case of CLL (SIR = 1.9). None of these estimates was significant.

### Discussion

The findings of this study, conducted in populations with high standards of ascertainment, diagnosis and certification of skin cancer (Levi *et al.*, 1995*b*), confirm that there is an excess of skin cancers (including BCC) following lymphoid neoplasms (NHL and CLL) and an excess of NHL following skin cancer. The relationship was observed for both SCC and BCC, but was somewhat stronger for SCC. A consistent pattern of risk was observed with CMM, too, but the data were too scanty for any significant result. The lower risk of skin cancer following CLL may be due to the low number of cases, or may indicate a reduced association compared with that observed with NHL.

Thus, the main contribution of this study is in showing that not only SCC and CMM, but also BCC (which is seldom registered in cancer registration schemes), is related to NHL and CLL. The associations were significant and consistent for BCC although somewhat less strong than for SCC.

Assuming that the underlying mechanism of the observed relationship between incidence of skin cancer and lymphoid neoplasms is U.V. radiation – and the consequent immunosupression of lymphoid neoplasms (Cartwright *et al.*, 1994; Hartge and Devesa, 1992; Doll, 1996) – this may reflect a differential role of U.V. exposure on the incidence of SCC and BCC. Data from the same population (Franceschi *et al.*, 1996) indicated that both histotypes were more common in frequently sun-exposed areas in both sexes, although the excess was greater for SCC. Other studies conducted in Denmark and Sweden (Adami *et al.*, 1995; Hall *et al.*, 1995)

**Table III** Observed (OBS) and expected (EXP) cases, and standardised incidence ratio (SIR) of subsequent non-Hodgkin's lymphoma and chronic lymphocyctic leukaemia (CLL) after an initial diagnosis of skin cancer in the cantons of Vaud and Neuchâtel, Switzerland, 1974–93

| | *Subsequent non-Hodgkin's lymphoma* | | | *Subsequent chronic lymphocytic leukaemia* | | |
|---|---|---|---|---|---|---|
| | *OBS* | *EXP* | *SIR (95% confidence interval)* | *OBS* | *EXP* | *SIR (95% confidence interval)* |
| Squamous cell carcinoma | | | | | | |
| Overall | 23 | 9.0 | 2.6 (1.6–3.8) | 4 | 2.7 | 1.5 (0.4–3.8) |
| By sex | | | | | | |
| Men | 11 | 6.0 | 1.8 (0.9–3.3) | 3 | 1.9 | 1.6 (0.3–4.7) |
| Women | 12 | 3.6 | 3.4 (1.7–5.9) | 1 | 1.1 | 0.93 (0.0–5.2) |
| By age at diagnosis (years) | | | | | | |
| <75 | 4 | 1.1 | 3.7 (1.0–9.6) | 2 | 0.18 | 11.1 (1.2–40.1) |
| ⩾75 | 19 | 7.9 | 2.4 (1.4–3.8) | 2 | 2.5 | 0.79 (0.1–2.8) |
| By years of follow-up | | | | | | |
| <5 | 13 | 5.6 | 2.3 (1.2–4.0) | 3 | 1.7 | 1.8 (0.4–5.2) |
| ⩾5 | 10 | 3.4 | 3.0 (1.4–5.4) | 1 | 1.0 | 0.97 (0.0–5.4) |
| Basal cell carcinoma | | | | | | |
| Overall | 43 | 22.5 | 1.9 (1.4–2.6) | 7 | 6.5 | 1.1 (0.4–2.2) |
| By sex | | | | | | |
| Men | 23 | 13.5 | 1.7 (1.1–2.5) | 5 | 4.0 | 1.2 (0.4–2.9) |
| Women | 20 | 9.9 | 2.0 (1.2–3.1) | 2 | 2.8 | 0.71 (0.1–2.6) |
| By age at diagnosis (years) | | | | | | |
| <75 | 21 | 4.9 | 4.3 (2.7–6.6) | 3 | 0.81 | 3.7 (0.7–10.8) |
| ⩾75 | 22 | 17.5 | 1.3 (0.8–1.9) | 4 | 5.7 | 0.71 (0.2–1.8) |
| By years of follow-up | | | | | | |
| <5 | 23 | 11.4 | 2.0 (1.3–3.0) | 4 | 3.2 | 1.3 (0.3–3.2) |
| ⩾5 | 20 | 11.1 | 1.8 (1.1–2.8) | 3 | 3.3 | 0.9 (0.2–2.7) |
| Malignant melanoma | | | | | | |
| Overall | 4 | 2.0 | 2.0 (0.5–5.0) | 1 | 0.53 | 1.9 (0.0–10.5) |

also showed a stronger association between NHL and SCC (with SIR of the order of 5) than for CMM (SIR around or below 2). The latter estimate was also consistent with that of a cohort of more than 6171 cases of NHL collected in various areas of North America and Europe (Travis *et al.*, 1993).

A common aetiological correlate, U.V. exposure or other factors causing immunosuppression (Cartwright *et al.*, 1994; Sasieni and Bataille, 1995), appears, therefore, to be a likely explanation of the association observed. This is also consistent with certain descriptive features of skin neoplasms and lymphomas, including their upward trends over the last few decades in most developed countries (Devesa and Fears, 1992; Hartge and Devesa, 1992; Carli *et al.*, 1994; Levi *et al.*, 1995*a*,*b*; Hjalgrim *et al.*, 1996) and geographic correlational studies in the UK (Bentham, 1996; McMichael and Giles, 1996), although not in the US (Hartge *et al.*, 1996). Moreover, there is a lack of individual-based information on any potential relationship between sun – and other sources of U.V. exposure – and the risk of lymphoid neoplasms. Before any such data are available, any inference on aetiological correlates should be considered speculative.

Increased surveillance and diagnosis following another neoplasm should also be considered. Although this could be a reasonable interpretation for at least part of the skin cancer excess following lymphoma, it is unlikely that a past history of SCC or BCC would materially modify subsequent ascertainment and diagnosis of lymphomas and CLL. Furthermore, no systematic excess of any cancer sites was observed: following skin melanoma the SIR for any site was 1.1 for men and 0.8 for women; following non-melanomas 1.0 for both; and following lymphomas 1.3 for men and 1.0 for women (Levi *et al.*, 1993).

The absence of any clear pattern of risk with increasing time after diagnosis of the first neoplasm is also incompatible with a major role of ascertainment or diagnostic bias. Among the other advantages of this study are its population basis, the inclusion of various histotypes of skin cancer, which should render any estimate relatively free from selection bias, and the complete histological confirmation of the various types of neoplasms considered.

In conclusion, therefore, this study on a population that was particularly well surveyed for skin cancers (Levi *et al.*, 1995*b*) confirms the presence of an association between SCC, NHL and, although less strongly, CLL. For the first time, these data also demonstrated an association between BCC, NHL and CLL, supporting the hypothesis that common aetiological factors are a determinant of the increased incidence of various types of skin and lymphoid neoplasms. The accumulating evidence on the issue has relevance to prevention

**Acknowledgements**
The contribution of the staff of the Swiss and Neuchâtel Leagues against Cancer and of the Vaud and Neuchâtel Cancer Registries is gratefully acknowledged.

### References

ADAMI J, FRISCH M, YUEN J, GLIMELIUS B AND MELBYE M. (1995). Evidence of an association between non-Hodgkin's lymphoma and skin cancer. *Br. Med. J.*, **310**, 1491–1495.

BENTHAM G. (1996). Association between incidence of non-Hodgkin's lymphoma and solar ultraviolet radiation in England. *Brit. Med. J.*, **312**, 1128–1131.

BRESLOW NE AND DAY NE. (1987). *Statistical Methods in Cancer Research. The Analysis of Case–control Studies.* vol. 2. IARC Scientific Publication no. 82: Lyon.

CARLI PM, BOUTRON MC, MAYNADIE M, BAILLY F, CAILLOT D AND PETRELLA T. (1994). Increase in the incidence of non-Hodgkin's lymphomas: evidence for a recent sharp increase in France independent of AIDS. *Br. J. Cancer*, **70**, 712–715.

CARTWRIGHT R, MCNALLY R AND STAINES A. (1994). The increasing incidence of non-Hodgkin's lymphoma (NHL): the possible role of sunlight. *Leukemia and Lymphoma*, **14**, 387–394.

DEVESA SS AND FEARS T. (1992). Non-Hodgkin's lymphoma time trends: United States and international data. *Cancer Res.* (suppl), **52**, 5432–5440.

DOLL R. (1996). Nature and nurture. Possibilities for cancer control. *Carcinogenesis*, **17**, 177–184.

FRANCESCHI S, LEVI F, RANDIMBISON L AND LA VECCHIA C. (1996). Site distribution of different types of skin cancer: new etiological clues. *Int. J. Cancer*, **67**, 24–28.

FRISCH M AND MELBYE M. (1995). New primary cancers after squamous cell skin cancer. *Am. J. Epidemiol.*, **141**, 916–922.

HALL P, ROSENDHAL I, MATTSSON A AND EINHORN S. (1995). Non-Hodgkin's lymphoma and skin malignancies – shared etiology? *Int. J. Cancer*, **62**, 519–522.

HARTGE P AND DEVESA SS. (1992). Quantification of the impact of known risk factors on time trends in NHL incidence. *Cancer Res.* (suppl.), **52**, 5566–5569.

HARTGE P, DEVESA S AND FRAUMENI JR J. (1994). Hodgkin's and non-Hodgkin's lymphomas. In *Trends in Cancer Incidence and Mortality*. Cancer Surveys, 19/20. Doll R, Fraumeni JF Jr and Muir CS. (eds). pp. 423–453. Cold Spring Harbor Laboratory Press: New York.

HARTGE P, DEVESA SS, GRAUMAN D, FEARS TR AND FRAUMENI JF JR. (1996). Non-Hodgkin's lymphoma and sunlight. *J. Natl Cancer Inst.*, **88**, 298–300.

HJALGRIM H, FRISCH M, BEGTRUP K AND MELBYE M. (1996). Recent increase in the incidence of non-Hodgkin's lymphoma among young men and women in Denmark. *Br. J. Cancer*, **73**, 951–954.

LEVI F, TE VC, RANDIMBISON L AND LA VECCHIA C. (1992). Statistics from the Registry of the Canton of Vaud, Switzerland, 1983–87. In *Cancer Incidence in Five Continents*. vol. VI. Parkin DM, Muir CS, Whelan SL, Gao YT, Ferlay J and Powell J. (eds). pp. 762–765. IARC Scientific Publication no. 120. IARC: Lyon.

LEVI F, RANDIMBISON L, TE VC, ROLLAND-PORTAL I, FRANCESCHI S AND LA VECCHIA C. (1993). Multiple primary cancers in the Vaud Cancer Registry, Switzerland, 1974–89. *Br. J. Cancer*, **67**, 391–395.

LEVI F, LA VECCHIA C, LUCCHINI F, TE VC AND FRANCESCHI S. (1995*a*). Mortality from non-Hodgkin's disease and other lymphomas in Europe, 1960–1990. *Oncology*, **52**, 93–96.

LEVI F, FRANCESCHI S, TE VC, RANDIMBISON L AND LA VECCHIA C. (1995*b*). Trends of skin cancer in the Canton of Vaud, 1976–92. *Br. J. Cancer*, **72**, 1047–1053.

MCMICHAEL AJ AND GILES GG. (1996). Have increases in solar ultraviolet exposure contributed to the rise in incidence of non-Hodgkin's lymphoma? *Br. J. Cancer*, **73**, 945–950.

PELLAUX S, LEVI F AND MEAN AM. (1992). Statistics from the Registry of the Canton of Neuchâtel, Switzerland, 1983–1987. In *Cancer Incidence in Five Continents*. vol. VI. Parkin DM, Muir CS, Whelan SL, Gao YT, Ferlay J and Powell J. (eds). pp. 754–757. IARC Scientific Publication no. 120. IARC: Lyon.

SASIENI P AND BATAILLE V. (1995). Non-Hodgkin's lymphoma and skin cancer. Ultraviolet light is an unlikely explanation for association. *Br. Med. J.*, **311**, 749.

SWERDLOW AJ, STORM HH AND SASIENI P. (1995). Risks of second primary malignancy in patients with cutaneous and ocular melanoma in Denmark, 1943–1989. *Int. J. Cancer*, **61**, 773–779.

TRAVIS LB, CURTIS RE, HANKEY BF AND FRAUMENI JR JF. (1992). Second cancers in patients with chronic lymphocytic leukemia. *J. Natl Cancer Inst.*, **84**, 1422–1427.

TRAVIS LB, CURTIS RE, GLIMELIUS B, HOLOWATY E, VAN LEEUWEN FE, LYNCH CF, ADAMI J, GOSPODAROWICZ M, WACHOLDER S, INSKIP P, TUCKER MA, FRAUMENI JR JF AND BOICE JD. (1993). Second cancers among long-term survivors of non-Hodgkin's lymphoma. *J. Natl Cancer Inst.*, **85**, 1932–1937.

WHO. (1976). *International Classification of Diseases for Oncology, ICD-O.* 1st edition. WHO: Geneva.